# Frequency of craniofacial pain in patients with ischemic heart disease

**DOI:** 10.4317/jced.53078

**Published:** 2017-01-01

**Authors:** Mahin Bakhshi, Rezvan Rezaei, Maryam Baharvand, Sedigheh Bakhtiari

**Affiliations:** 1 Associate Professor, Dept. of Oral Medicine, Dental School, Shahid Beheshti University of Medical Sciences, Tehran, Iran; 2 Resident of Pediatric Dentistry, Dental School, Shahid Beheshti University of Medical Sciences, Tehran, Iran; 3 Professor, Dept. of Oral Medicine, Dental School, Shahid Beheshti University of Medical Sciences, Tehran, Iran

## Abstract

**Background:**

Referred craniofacial pain of cardiac origin might be the only symptom of ischemic heart accidents. This study aimed to determine the frequency of craniofacial pain in patients with ischemic heart disease.

**Material and Methods:**

This cross-sectional study was accomplished on 296 patients who met the criteria of having ischemic heart disease. Data regarding demographics, medical history and referred craniofacial pain were recorded in data forms. In addition, patients underwent oral examination to preclude any source of dental origin. Chi-square test, Student’s t-test and backward regression model were used to analyze the data by means of SPSS software version 21. *P*<0.05 was considered significant.

**Results:**

A total of 296 patients were studied comprising of 211 men (71%) and 85 women (29%) with the mean age of 55.8. Craniofacial pain was experienced by 53 patients out of 296, 35 (66%) of whom were male and 18 (34%) were female. None of the patients experienced craniofacial pain solely. The most common sites of craniofacial pain were occipital and posterior neck (52.8%), head (43.3%), throat and anterior neck (41.5%) respectively. We found no relationship between craniofacial pain of cardiac origin with age, diabetes, hypertension, and family history. On the other hand, there was a significant relationship between hyperlipidemia and smoking with craniofacial pain of cardiac origin.

**Conclusions:**

Radiating pain to face and head can be expected quite commonly during a cardiac ischemic event. Dental practitioners should be thoroughly aware of this symptomatology to prevent misdirected dental treatment and delay of medical care.

** Key words:**Craniofacial pain, ischemic heart disease, myocardial infarction, angina pectoris, referred pain.

## Introduction

Ischemic heart disease is considered as a major cause of death in adults (1). The cardinal symptom of ischemic heart disease is chest pain characteristically induced by activities such as walking, climbing stairs, eating, or stress. Convergence of vagus, trigeminal, and cervical (C2, C3) nerves, may cause the pain radiate to other areas like right or left shoulder, scapular region, neck and lower jaw (2-6). In rare occasions, pain is solely perceived in the aforementioned areas instead of the chest, which leads to increased mortality due to misdiagnosis. Kreiner *et al.* (6) reported the occurrence of craniofacial pain during ischemic heart accidents to be nearly 40%, which was the sole symptom of ischemic heart disease in 6% of cases. Ischemic heart disease can be manifested as angina pectoris and myocardial infarction (MI), with the former having two clinical types of stable and unstable angina. Patients with stable angina has a good prognosis, whereas those with unstable angina experience episodes of chest pain even in the rest position and are likely to progress to MI soon (7).

Pain of dental, periodontal, sinus and musculoskeletal origins are amongst the most common types of orofacial pain. However, pain in these areas might be originated from other regions, which are referred to as heterotopic pain (5). Cardiac pain can be presented as heterotopic pain, where in the orofacial region leads to unnecessary dental procedures and delay in diagnosis and treatment of cardiac disease. There are several reports regarding inappropriate dental treatment due to misdiagnosis of pain source (8). On the other hand, in developed countries missed diagnosis of MI has been described in 2-27% of cases (9,10). As demonstrated in a study, one fourth of misdiagnosis of MI resulted in lethal or potentially lethal complications (8) lack of chest pain and slow twitch (ST) elevation being the most important causes (10). Patients suspected of acute myocardial infarction (AMI) with no chest pain have a three times higher risk of death compared with those having chest pain (11). Another study revealed that in the absence of chest pain, one-year mortality rate of cardiac patients was twice as high as those experiencing chest pains. Orofacial pain was recorded as the sole symptom of ischemic heart disease in 6% of patients, while 32% had orofacial pain accompanied with pain in other regions. The frequency of craniofacial pain of cardiac origin is higher in women than in men as shown in two studies (12,7).

Many studies have been conducted about frequency of cardiac pain in different parts of body (13-18), however few studies addressed referral of cardiac pain to head and neck areas with most of them being case reports (19-27). Therefore, this study aimed to determine the frequency of craniofacial pain in patients with ischemic heart disease.

## Material and Methods

This cross-sectional study was accomplished on 296 hospitalized patients who met the criteria of having ischemic heart disease (angina pectoris or myocardial infarction) verified by means of angiography in Shahid Rajaie Cardiovascular, Medical & Rese-arch Center, Tehran, Iran. History of previous cardiovascular disease such as hospitalization in coronary care unit (CCU), consumption of cardiovascular medications, history of severe chest pain as well as risk factors of coronary heart disease like diabetes mellitus, hypertension, smoking, hyperlipidemia, and family history of heart disease were all recorded in data forms. Patients with history of chronic headache, earache, severe psychiatric disorders, pain in the tempororomandibular joint (TMJ) region, surgery or presence of a diagnosed mass in the jaws, and recent odontogenic pain were excluded from the study. Meanwhile, all eligible patients underwent oral examination by means of dental mirror and flashlight while lying on their beds, and those who found to have dental problems (severe or complex dental caries and any suspected tooth with pulp exposure) were banned from entering the study.

**Figure 1:**
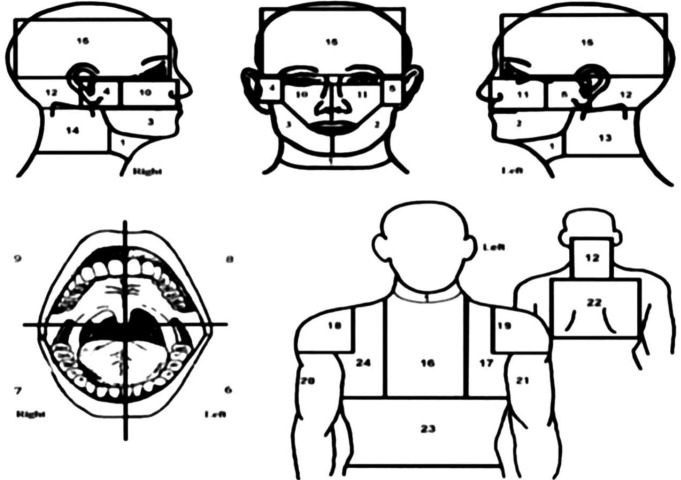
Figure of the body and the craniofacial structures subdivided into different areas.

On the day after angiography, patients were fully informed about details of the study, and then requested to fill out the data forms, which was sub divided into two part, providing demographics and pain characteristics (head and neck pain before or during heart attack, pain in other parts of body, seeking or receiving dental care due to craniofacial pain). Thereafter patients were shown anatomic illustrations representing chest, abdomen, back, shoulders, arms, face, neck, and mouth, and asked to mark site of their pain on the picture (6) (Fig. 1). To ensure the acuity of data, all questionnaires were reviewed by the researcher, and patients were asked once more regarding having pain and pointing to site of pain.

The study protocol was approved by oral medicine department of Shahid Beheshti University of Medical Sciences and Shahid Rajaie Cardiovascular, Medical & Research Center. All patients were obtained informed consent to participate in the study.

-Data analysis:

To analyze the data, SPSS software version 21 was used. Descriptive statistics was used to report the results. Moreover, Chi-square test was used to examine differences between two genders in terms of symptoms, and Student’s t-test to assess the distribution of age between two groups (with pain and without pain). In order to analyze the effect of gender, age, type of cardiac disease, and coronary risk factors on chance of having craniofacial pain, backward regression model was utilized. *P*<0.05 was considered significant.

## Results

The present study was aimed to determine the frequency of craniofacial pain of cardiac origin in patients with ischemic heart disease hospitalized in Shahid Rajaie Cardiovascular, Medical & Research Center in the year 2014-2015. A Total of 296 patients entered the study comprising of 211 men (71%) and 85 women (29%) age ranging 29 to 85 years with the mean age of 55.8. Of the total sample, 133 patients were hospitalized for MI, 71 for unstable angina, and 92 for stable angina. In regard to medical records, 166 patients had history of hospitalization in CCU, 209 were taking cardiovascular medications, and 150 experienced severe chest pain. Meanwhile, medical history taking revealed diabetes in 95 patients, hypertension in 134, smoking in 106, and hyperlipidemia in 148 and presence of ischemic heart disease in first-degree relatives in 149.

Craniofacial pain was experienced by 53 patients out of 296, 35 (66%) of whom were male and 18 (34%) were female. The age range of men was 57.9 and that of women was 62.8. Thirty six patients (66%) were hospitalized because of MI, and 17(34%) for unstable angina.

**Figure 2:**
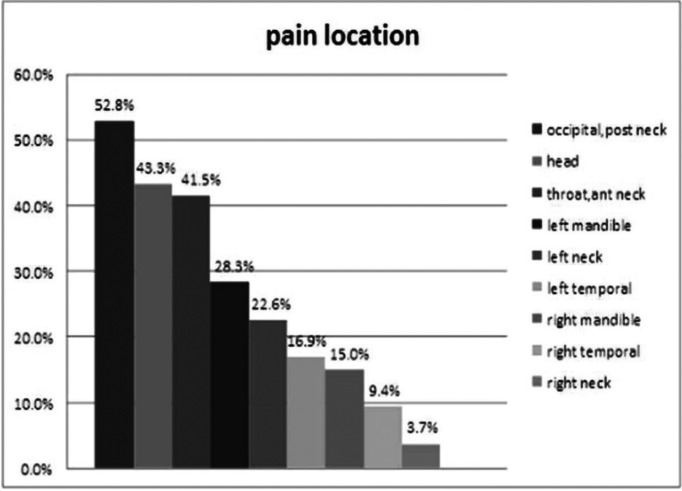
Distribution of pain location.

None of the patients experienced craniofacial pain solely, but all had concomitant pain in mid-chest or left chest. In case of craniofacial pain, the most common sites of pain were occipital and posterior neck (52.8%), head (43.3%), throat and anterior neck (41.5%) respectively. Pain in the left mandible was recorded in 28.3% of patients. The right neck was the least frequently site of referred craniofacial pain (3. 7%) (Fig. 2).

There was no report of referral pain in the maxilla or teeth among our patients.

The most prevalent site of pain in patients without history of craniofacial pain were mid-chest, left side of the chest, back, and left shoulder. On the other hand, those with craniofacial pain had cardiac pain most commonly in left side of the chest, mid-chest, and back respectively.

We found no relationship between age and craniofacial pain of cardiac origin. However, there was a statistically significant difference between men and women with craniofacial pain in terms of sex (*p*=0.02).

**Figure 3:**
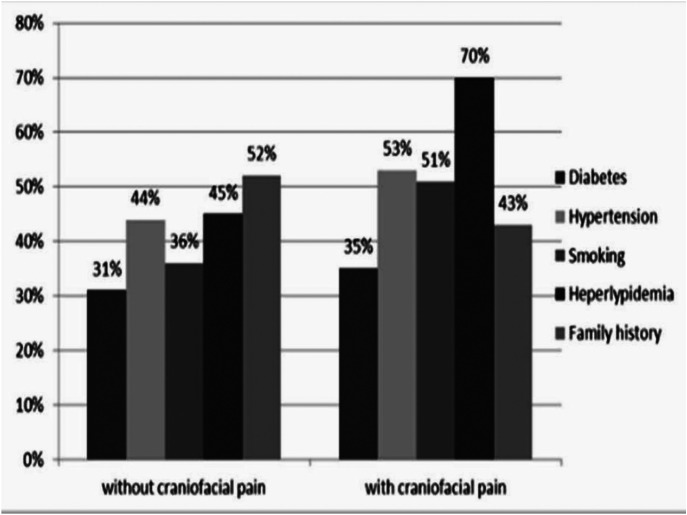
Coronary risk factors in patients with and without craniofacial pain.

Meanwhile, no association was detected between craniofacial pain of cardiac origin with diabetes, hypertension, and family history. There was a significant relationship between hyperlipidemia (*p*=.001) and smoking (*p*=.003) with craniofacial pain of cardiac origin (Fig 3).

## Discussion

Ischemic heart disease is considered one of the major fatal events among adults (1). Patients with ischemic heart disease may experience referred pain in the head and neck areas. Induction of pain radiation after physical activity and relief following rest is indicative of cardiac origin (7). There are a few studies regarding frequency of cardiac pain referred to head and neck in patients with ischemic heart disease. The objective of the present study was to determine the frequency of craniofacial pain of cardiac origin in patients hospitalized at Shahid Rajaie Cardiovascular, Medical & Research Center.

In this study patients were selected after confirmation of having severe coronary stenosis based on angiographic interpretation by an experienced cardiologist. In other similar studies the method of patient selection was not mentioned (27,28).

Danesh-Sani *et al.* (28) found 34% of patients having craniofacial pain of cardiac origin, which was the only presentation of i-chemic heart disease in 13.3% of them. In Kreiner *et al.* (27) study, these values were 38% and 15% respectively. In the present study, 17.9% of patients reported to have referred craniofacial pain accompanied by pain in other parts of the body, and none of them presented with craniofacial pain as the sole symptom during heart attack. Due to low public awareness of craniofacial pain as a symptom of cardiac ischemia, the frequency found in our study regarding craniofacial pain of cardiac origin is likely to cons-titute an underestimation.

In accordance to our results, there was no association between age and craniofacial pain in similar studies (*p*=0.2). We found a significant difference between men and women with craniofacial pain with respect to age in a way that mean age of women was significantly more than of men (*p*=0.025).

Previous studies have demonstrated significant differences between men and women in terms of referred pain to craniofacial region. Kreiner *et al.* (27) and Danesh-Sani *et al.* (28) showed that women experienced craniofacial pain more frequently than did men. Danesh-Sani *et al.* (28) reported significantly higher frequency of craniofacial pain among men compared to women. In the present study, there was no significant difference between men and women in regard to craniofacial pain (*p*=0.352).

Kreiner *et al.* (27) noticed that the most common sites of craniofacial pain were upper throat and anterior neck (81.7%), left mandible (45.1%), and right mandible (40%). However, Danesh-Sani *et al.* (28) found left mandible as the most common site of involvement with referred craniofacial pain . In this study, regions of occipital and back neck (52.8%), head (43.3%), and anterior neck and throat (41.5%) were found to be the most prevalent sites of craniofacial pain. Pain in the left mandible was perceived by 28.3% of our patients, and there was a significant difference between left and right mandible in this regard in a way that left mandible was significantly affected higher than right mandible (*p*=0.02).

Contrary to our results, Danesh-Sani *et al.* (28) and Kreiner *et al.* (29) reported that the most common site of pain in the absence of chest pain was maxillofacial region. In this study, patients without chest pain reported regions of back and low back as the most prevalent sites of pain.

We were not able to take panoramic views of hospitalized patients. In addition, patients examined by means of observation with dental mirror in supine position. Danesh-Sani *et al.* (28) ordered panoramic views for all patients, which might have excluded more patients due to having non-cardiac pain.

It is noteworthy that the previous studies neither addressed craniofacial pain after angiography, nor considered coronary risk factors such as diabetes, hypertension, hyperlipidemia, smoking, and family history. Our study found no relationships between craniofacial pain of cardiac origin with diabetes, hypertension, and family history. However, there was a significant relationship between craniofacial pain with hyperlipidemia (*p*=0.01) and smoking (*p*=0.03).

In Kreiner *et al.* (6) study, three cardiac patients experienced bilateral toothache in the mandible, and one had left maxillary odontogenic pain. In addition, the ratio of bilateral craniofacial pain to unilateral pain was 6:1, whereas this ratio was 1:1 in the arms. However there was no report of referral pain in the maxilla or teeth among in present study.

Regarding the crucial risk of ischemic heart disease and possibility of pain referral solely to the craniofacial area, it is recommended that further studies be conducted with larger sample size, more elaborate oral examination including radiographic imaging as well as detailed inspection of facial, muscular, and temporomandibular structures, and recording pattern of pain.

Radiating pain to face and head areas can be expected quite commonly during a cardiac ischemic event. Since dental practitioners may play an important role to detect such atypical symptoms of cardiac origin; they should be thoroughly aware of this symptomatology in order to prevent misdirected dental treatment and delay of medical care.
